# A comprehensive review of clear cell stromal tumor of the lung: integrating clinicopathology and molecular pathogenesis

**DOI:** 10.3389/fonc.2026.1760048

**Published:** 2026-02-10

**Authors:** Liping Luo, Liang Lv, Ying Zeng

**Affiliations:** 1 Department of Pathology, Daping Hospital, Amy Medical University, Chongqing, China; 2 Department of Pathology, Chongqing Public Health Medical Center, Chongqing, China; 3 Department of Pathology, The Thirteenth People’s Hospital of Chongqing, Chongqing, China

**Keywords:** clear cell stromal tumor of the lung, clinicopathology, molecular pathogenesis, treatment and prognosis, *YAP1::TFE3* gene fusion

## Abstract

Clear cell stromal tumor (CCST) is a newly recognized mesenchymal neoplasm of the lung, with a recurrent *YAP1::TFE3* gene fusion in most cases and characterized histologically by spindle, epithelioid cells, with variably clear to pale eosinophilic cytoplasm and prominent vascularity. Due to its rarity and the absence of specific immunohistochemical markers beyond TFE3, diagnosis remains challenging. Currently, only thirty cases have been documented. This narrative literature review synthesizes current advances to enhance understanding of this entity.

## 1 Introduction

Clear cell stromal tumor of the lung (CCST-L) is a rare primary mesenchymal pulmonary neoplasm of unknown histogenesis, with an indeterminate incidence among all lung tumors since its first description in 2013 (1). CCST-L exhibits non-specific clinical and radiological features. Definitive diagnosis requires integration of histopathological, immunohistochemical (IHC), and molecular analyses. Histologically, CCST-L is characterized by tumor cells with clear to eosinophilic cytoplasm, cytological blandness, and low-grade nuclear features. Cytomorphologically, the tumors were mostly composed of histiocytoid, ovoid-to-spindled cells. The neoplasm demonstrates scant mitotic activity (<1 mitotic figure per 2mm^2^) and thin-walled and/or staghorn shaped vessels. Immunophenotypically, it shows diffuse strong positivity for Vimentin and TFE3 but is negative for lineage-specific markers (e.g., myoepithelial, alveolar epithelial, neuroendocrine, squamous, melanocytic, neural, and vascular). Molecular analysis reveals *YAP1::TFE3* gene fusion in most cases, serving as a key marker. Given its rarity, this review details CCST-L’s clinicopathological and molecular characteristics to facilitate accurate diagnosis by pathologists and clinicians.

## 2 Methods

This study is structured as a narrative review aiming to synthesize the current knowledge and evidence on clear cell stromal tumor of the lung (CCST-L). The primary objective is to provide a comprehensive overview of its clinical presentation, pathological and molecular features, diagnostic challenges, management approaches, and prognosis, based on the available published literature.

To identify relevant literature, we conducted a broad and comprehensive survey of published articles. Electronic databases, including PubMed/MEDLINE, Web of Science, and Wanfang Database (China), were searched up to April 2025. The search was not restricted by publication date or language. Key terms and their combinations used in the search included: “clear cell stromal tumor of the lung,” “CCST-L,” “Hemangioblastoma-like Clear Cell Stromal Tumor of the Lung,” “TFE3,” “*YAP1::TFE3* fusion,” “*YAP1-TFE3*,” and “primary pulmonary clear cell sarcoma.” Reference lists of identified articles were also manually reviewed to capture additional pertinent sources.

### 2.1 Literature selection and synthesis approach

Given the rarity of CCST-L, our goal was to be inclusive of all available case reports, small case series, and relevant review articles. A formal systematic review protocol with predefined inclusion/exclusion criteria and a dual-independent screening process was not employed, as this work is intended as a narrative synthesis. Articles were selected based on their direct relevance to the clinicopathological description, molecular genetics, diagnosis, or management of CCST-L. Findings from the identified literature were then thematically analyzed and integrated to construct a coherent summary of the entity. Special attention was paid to reconciling consistent findings across studies while also noting areas of discrepancy or limited evidence. The strength of evidence for different conclusions is explicitly discussed in the relevant sections of the manuscript.

## 3 Results

This search yielded 11 eligible publications (1–11). All cases underwent rigorous evaluation with data extraction encompassing: demographics (age, sex), clinical presentation, radiological features, *YAP1::TFE3* fusion status, treatment modalities, and clinical outcomes, which were summarized in **Table 1**.

**Table 1:** summarizes the clinicopathological features of lung clear cell stromal tumors.

References	Case	Age(Years)	Gender	Symptoms	The size(cm) and location of the mass	Molecular detection	Surgery/treatment	Lymph node status/metastasis situation	Follow-up time/result
Falconieri et al, 2013 (1)	1	68	female	Mild fever,flu-like symptoms	6/Right upper lobe of the lung	Not detected	Lobectomy	(-)	24 months/No recurrence
	2	40	male	NA	3/Left lower lobe of the lung	No mutations in the coding sequence of the *VHL* gene	Lobectomy	(-)	24 months/No recurrence
Lindholm et al, 2020 (2)	3	46	female	Dyspnea	2.8/Right lower lobe of the lung	Not detected	Lobectomy	NA	36 months/No recurrence
	4	47	female	Dyspnea	2/Right upper lobe of the lung	Not detected	Lobectomy	NA	20 months/No recurrence
	5	52	male	Cough	2.5/Left upper lobe of the lung	Not detected	Lobectomy	NA	NA
	6	39	female	Dyspnea	2.5/Right upper lobe of the lung	Not detected	Lobectomy	NA	18 months/No recurrence
	7	42	female	Cough,chest pain	2/Left lower lobe of the lung	Not detected	Lobectomy	NA	NA
Agaimy et al, 2021 (3)	8	56	female	Hemoptysis	2.3/Left upper lobe of the lung	*YAP1*(exon4)::*TFE3*(exon7)	Lobectomy	(-)	36 months/No recurrence
	9	29	female	Hemoptysis	9.5/Lung lobe	*YAP1*(exon5)::*TFE3*(intron6)	Biopsy	Both kidneys and the liver are involved	NA
	10	69	female	NA	4/Left upper lobe of the lung	YAP1(exon4)::TFE3(exon7)	Lobectomy	Hilar lymph nodes(+)	12 months/No recurrence
	11	66	female	NA	NA/Left main bronchus	No fusion of the *YAP1::TFE3* gene, a *STED2* mutation	Biopsy	(-)	4 years/Disease progression
Dermawan et al, 2021 (4)	12	77	male	NA	3.9/Left lower lobe of the lung	*YAP1*(exon4)::*TFE3*(exon7)	Wedge resection	(-)	7 months/No recurrence
	13	35	male	NA	7.5/Middle lobe of the right lung	*YAP1*(exon4)::*TFE3*(exon7)	Wedge resection	(-)	6 months/No recurrence
Zhang et al., 2022 (5)	14	40	female	Found during physical examination	0.8/Right upper lobe of the lung	No fusion of the *YAP1::TFE3* gene	Lobectomy	(-)	3 months/No recurrence
Dehner et al, 2022 (6)	15	24	female	Dyspnea,chest pain	5.8/Multiple lesions in both lungs	*YAP1*(exon4)::*TFE3*(exon7)	Biopsy,Chemotherapy	Lymph node(+)	7 months/death
	16	55	female	Severe chest pain	9.5/Left upper lobe of the lung	*YAP1*(exon4)::*TFE3*(exon7)	Lobectomy	(-)	43 months/No recurrence
	17	66	female	NA	2.9/Middle lobe of the right lung	*YAP1*(exon4)::*TFE3*(exon7)	Biopsy	(-)	NA
	18	69	female	NA	1.0/Right lower lobe of the lung	*YAP1*(exon4)::*TFE3*(exon7)	Wedge resection	(-)	NA
Jakša et al., 2023 (7)	19	57	male	NA	4.5/Right upper lobe of the lung	*YAP1*(exon4)::*TFE3*(exon7)	Lobectomy	(-)	12 months/No recurrence
Weissferdt et al.,2024 (8)	20	58	female	Incidental finding; SSA (Ro) autoantibodypositive	3/Multiple lesions in both lungs	Not detected	Lobectomy	(-)	26 months/Disease progression
	21	56	male	Incidental finding; seronegative rheumatoid arthritis	2.5/Multiple lesions in both lungs	*YAP1*(exon4)::*TFE3*(exon7)	Lobectomy,Chemotherapy	(-)	66 months/Disease progression
	22	47	male	shortness of breath,chest pain	NA/Multiple lesions in both lungs	Not detected	Lobectomy	(-)	24 months/Disease progression
Huang et al.,2024 (9)	23	55	female	shortness of breath	3.2/Left lower lobe of the lung	*YAP1*(exon4)::*TFE3*(exon7)	Lobectomy	(-)	12 months/No recurrence
Odintsov et al.,2025 (10)	24	42	male	NA	2.3/Left lower lobe of the lung	*YAP1*(exon4)::*TFE3*(exon7)	Lobectomy	(-)	104 months/No recurrence
	25	56	female	NA	NA/Right lung	*YAP1*(exon4)::*TFE3*(exon7)	Lobectomy	(-)	NA
	26	63	male	NA	2/Right lower lobe of the lung	*YAP1*(exon4)::*TFE3*(exon7)	Lobectomy	(-)	3 months/No recurrence
	27	84	female	NA	5.5/Left lower lobe of the lung	*YAP1*(exon4)::*TFE3*(exon7)	Lobectomy	(-)	18 months/No recurrence
	28	61	female	NA	0.7/Right lower lobe of the lung	*YAP1*(exon4)::*TFE3*(exon7)	Lobectomy	(-)	3 months/No recurrence
	29	41	male	NA	3.4/Right upper lobe of the lung	*TFE3* rearrangement	Lobectomy	(-)	22 months/No recurrence
Jian Zeng et al.2025 (11)	30	53	male	cerebral infraction, incidentally discover	2/left lower lobe of the lung	No fusion of the *YAP1::TFE3* gene	Lobectomy	(-)	NA/No recurrence or distant metastasis.

NA, Not available.

### 3.1 Clinical features

CCST-L is exceptionally rare, with only 30 pathologically confirmed cases documented to date (1–11). This neoplasm primarily arises in the lung parenchyma with frequent bronchial involvement. Epidemiologically, the median age at diagnosis is 53 years (range: 24–84 years) with a marked female predominance (female-to-male ratio=19:11). Tumor location was as follows: right upper lobe (n=5), right middle lobe (n=2), right lower lobe (n=4), left upper lobe (n=4), left lower lobe (n=8), bilateral lungs (n=4), and left main bronchus (n=1).Two cases lacked precise localization. Clinical symptom data were available for 16 patients. Symptomatic presentations (12/16, 75%) included cough, hemoptysis, dyspnea, chest tightness chest pain, shortness of breath, and fever. Four patients were asymptomatic, with tumors detected incidentally during routine imaging. Medical history included seronegative rheumatoid arthritis (n=1) (8)and cerebral infarction (n=1) (11). Despite established links between tobacco and pulmonary malignancies, no smoking history was documented in any reported CCST-L patient. Comprehensive clinical characteristics are presented in **Table 1**.

### 3.2 Imaging findings

Chest computed tomography (CT) is the primary imaging modality for evaluating CCST-L, revealing features that, while non-specific, can suggest the diagnosis within an appropriate clinical context. The findings can be systematically categorized as follows:

#### 3.2.1 Consistent patterns

The most frequently reported pattern is a single, well-defined, expansile soft-tissue mass with intraparenchymal growth, often showing close association with or involvement of segmental or mainstem bronchi.

#### 3.2.2 Spectrum of variability

The radiographic presentation exhibits a significant range.

(i) Tumor Burden: While most cases are solitary, multifocal or bilateral pulmonary nodules/masses have been documented in several patients, immediately raising the differential of metastatic disease (6, 8).
(ii) Metabolic Activity: Fluorodeoxyglucose positron emission tomography (FDG-PET) data are limited but available cases have shown marked hypermetabolism, indicative of high glucose avidity (6).
(iii) Aggressive Features: Imaging may reveal findings associated with worse prognosis, including lymph node enlargement at diagnosis (6).

#### 3.2.3 Diagnostic implications and challenges

Preoperatively, the non-specific radiologic appearance of CCST-L frequently leads to initial interpretations suggestive of more common entities such as infection (pneumonia), benign lesions, or other mesenchymal tumors (e.g., synovial sarcoma, PEComa). This underscores that imaging cannot reliably differentiate CCST-L from its mimics; its primary role lies in defining disease extent (localized vs. multifocal) and identifying features (e.g., nodal disease) that correlate with aggressive behavior, thereby guiding clinical management.

### 3.3 Pathological features

Gross examination revealed unencapsulated but well-demarcated tumors with a grayish-white to tan-yellow cut surface. The median tumor size was 5.1 cm (range: 0.7-9.5 cm), demonstrating solid and cystic components with cavernous architecture and friable consistency. Texture varied from medium to firm (8). Most tumors arose within the pulmonary parenchyma, while three involved main or segmental bronchi. Synchronous bilateral lesions were observed in four patients.

**Figure 1:**
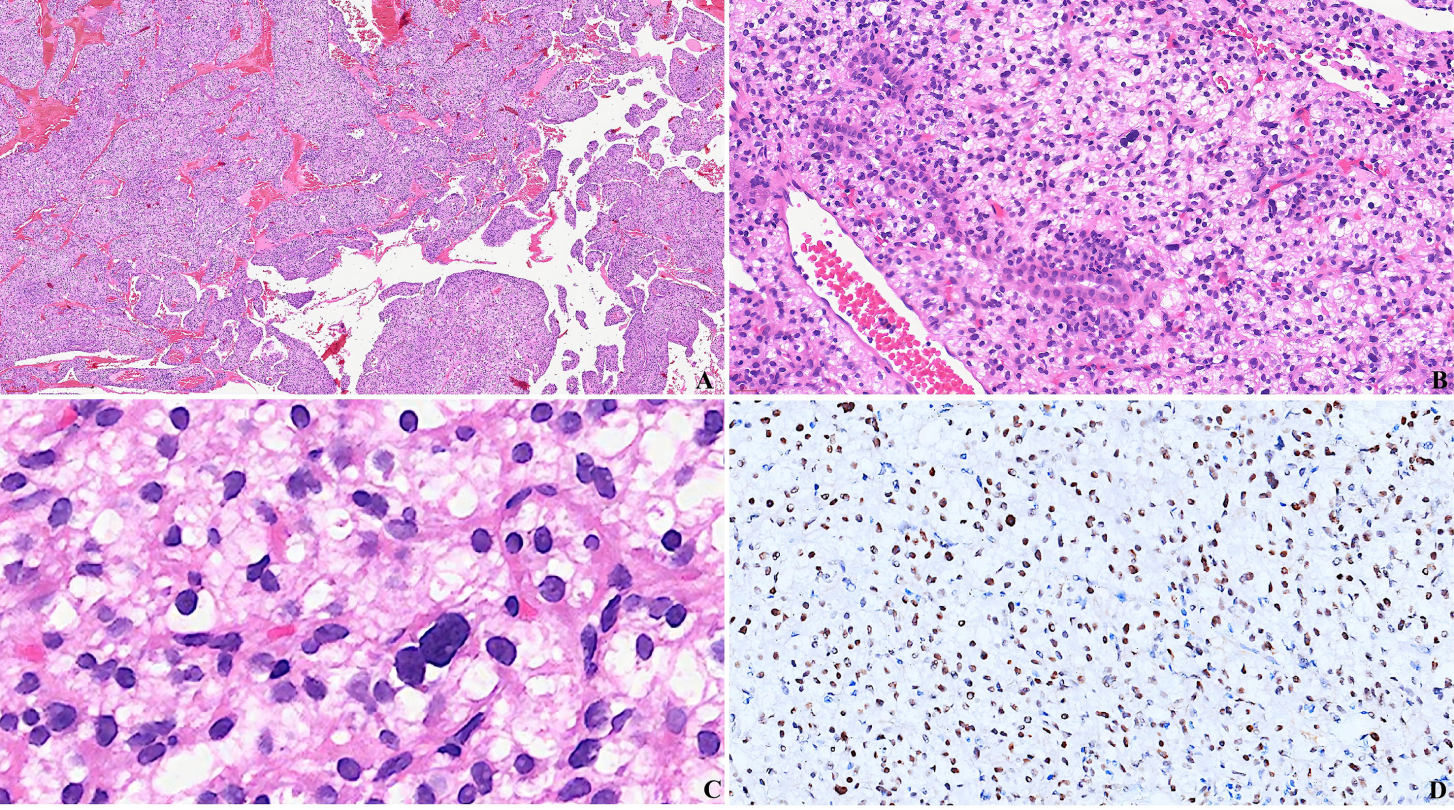
Pathological Characteristics of Clear Cell Stromal Tumor of the Lung. A-C Photomicrograph of the tumor (H&E stain). **(A)** The tumor demonstrates an arrangement of solid sheets, nests, or trabecular structures, compartmentalized by sinusoids lined with delicate vascular walls(H&E stain, Scale bar: 200 μm). **(B)** The tumor cells exhibit epithelioid morphology with abundant clear or eosinophilic cytoplasm. Foci of residual normal alveolar epithelium are occasionally observed within the tumor cell nests. (H&E stain, Scale bar: 100μm). **(C)** Higher-magnification showed the neoplastic nuclei demonstrate bland cytological features characterized by fine chromatin and inconspicuous nucleoli. Occasional nuclear pleomorphism may be noted. Mitotic figures are rare(H&E stain, Scale bar: 50μm). **(D)** Photomicrograph of immunohistochemical staining. Immunohistochemical analysis demonstrates strong nuclear positivity for TFE3 in the tumor cells.(IHC stain, Scale bar: 100 μm).(These images were kindly provided by Professor Zhao Ming (9) from the Ningbo Clinical Pathological Diagnosis Center, China).

Microscopically, the tumors demonstrated solid cellular proliferation with subtle nested architecture alternating with prominent, thin-walled dilated vessels exhibiting staghorn configurations at low power(**Figure 1A**). At intermediate magnification, neoplastic cells effaced native lung parenchyma with scattered entrapped bronchial glands(**Figure 1B**). High-power examination revealed proliferations of spindle, oval, epithelioid, or polygonal cells exhibiting indistinct cell borders, mild to moderate nuclear atypia, eccentrically placed hyperchromatic nuclei (round/oval/spindle), generally inconspicuous nucleoli, abundant clear-to-eosinophilic cytoplasm(**Figure 1C**). Notably, the “clear cell” designation belied significant cytomorphologic heterogeneity, with variable proportions of cells showing clear, pale histiocytoid, or eosinophilic features. The stroma contained scattered foamy histiocytes, mixed inflammatory cells (lymphocytes, plasma cells, eosinophils), and focal calcifications. Periodic Acid-Schiff stain(PAS) staining revealed diastase-sensitive cytoplasmic glycogen deposits (3), supporting clear cell morphology.

Beyond core morphology, CCST-L exhibits several uncommon features: (i) Adipocytic Differentiation & Stromal Changes: scattered adipocytes and focal adipose-rich areas, liponecrotic changes, peritumoral lymphoid aggregates, tumor regression within fibrous, scar-like stroma (1), discrete hyalinization in 5 cases (2). (ii) Cytoplasmic and nuclear variations: predominantly eosinophilic cytoplasm and without clear cytoplasm (4), degenerative nuclear changes: scattered pleomorphic cells with hyperchromatic, lobulated nuclei in 2 cases (3). (iii) Necrosis and vascular abnormalities: ischemic-type necrosis with hemorrhagic foci in 2 cases (3), absence of coagulative necrosis (3), intratumoral vascular thrombosis in 5/8 cases (10). (iv) Aggressive phenotype: geographic necrosis, bizarre nuclei and elevated mitotic figure (6 per 2 mm²) (6).

### 3.4 Immunohistochemistry findings

Given CCST-L’s rarity, definitive diagnosis requires integration of immunohistochemistry (IHC) and molecular testing. Among 30 confirmed cases, immunohistochemical analysis demonstrates strong nuclear positivity for TFE3 (**Figure 1D**) in 22/23 cases (95%) tested since 2021 (7cases before 2021 lacked testing). Vimentin showed uniform diffuse positivity in all cases. CD34 was focally expressed in only 2 case (5, 7). Consistent negative markers (all cases) were CK, EMA, TTF-1, p40, CD56, SYN, CD31, ERG, CK7, S100, HMB45, Melan-A, α-inhibin, CD45, CD23, GFAP, Desmin, SMA and STAT6[5]. Rare/limited expressions: focal calponin and h-caldesmon (1 case) (5), isolated HMB45/MART1+ cells with negative MITF (3), weak patchy SMA (exceptional).

### 3.5 Molecular findings

Molecular studies have consistently identified the *YAP1::TFE3* gene fusion frequently observed in CCST-L. Among 21 cases which molecular testing was performed, FISH confirmed the fusion in 18 cases (86%) and failed to detect a fusion in three cases(14%) (3, 5, 11) despite their typical histology. Notably, the fusion-negative case showed TFE3 expression that is comparable to the reactivity seen in the fusion-positive cases suggesting possible activation or overexpression via other unknown mechanisms (3). RNA sequencing detected *YAP1*(exon4)::*TFE*3(exon7) fusions in 17 cases (81%) and *YAP*1(exon5)::*TFE3*(intron6) in one case (5%). In the *YAP1* exon 4::*TFE3* exon 7 fusion, YAP1 contributes the TEAD interacting domain (TID), WW domains, and 14-3–3 protein binding site, whereas the TFE3 portion adds the Basic Helix-Loop-Helix (bHLH) domain, Leucine zipper domain and the C-terminal transactivation domain. Thus, the resulting chimeric oncoprotein likely draws on the strength of both parent proteins and may evade some of the physiological negative regulation (10). Of note, the *YAP1*::*TFE3* rearrangement that occurs in the variant epithelioid hemangioendothelioma (EHE) connects *YAP1* exon 1 to *TFE3* exon 4 or 6 (12). Unlike the fusion in CCST-L, this rearrangement lacks the 14-3–3 binding site S127. When phosphorylated, S127 creates a binding site for 14-3–3 that sequesters YAP1 to the cytoplasm and suppresses its nuclear activity (13). Retained regulation by 14-3–3 might explain the relatively indolent clinical course of CCST-L. Notably, TFE3 IHC expression was consistent in *YAP1::TFE3* fusion-negative cases. Next-generation sequencing identified an *STED2* mutation in one case, suggesting alternative oncogenic pathways suggesting the potential existence of other unknown activation pathways in CCST-L (3). First described in 2013 as “pulmonary hemangioblastoma-like clear cell stromal tumor” due to morphological overlap, this entity was reclassified as CCST-L following discovery of *YAP1::TFE3* fusions, which distinguish it immunophenotypically from true hemangioblastomas.

### 3.6 Differential diagnosis

Although characterized by diffuse TFE3 expression, uniform vimentin positivity, and *YAP1::TFE3* fusion, the accurate diagnosis of CCST-L remains critically dependent on careful histological and immunophenotypic distinction from several key mimics. The differential diagnosis broadens considerably in patients presenting with multifocal disease, where the primary consideration shifts to excluding metastatic malignancy. A systematic approach is essential for accurate classification. The major entities to consider and their defining features are summarized in **Table 2**. The diagnostic process relies heavily on a combination of characteristic morphology, a judiciously applied immunohistochemical (IHC) panel, and ultimately, confirmatory molecular testing.

**Table 2:** Key differential diagnoses of clear cell stromal tumour of the lung (CCST-L).

Diagnostic entity	Key histological features	Characteristic immunophenotype (positive markers)	Negative/crucial discriminatory markers	Molecular alterations	Key distinguishing features & notes
Clear Cell Stromal Tumour of the Lung (CCST-L)	Sheets/nests of clear/eosinophilic cells within a sinusoidal vascular network; low mitotic activity.	TFE3 (nuclear+), Vimentin (diffuse strong+); YAP1-CT loss with TFE3 overexpression is a specific surrogate pattern.	HMB45-, Melan-A-, SMA-, STAT6-, CD31-, ERG-, TTF-1-, PAX8-, EMA-.	*YAP1::TFE3* gene fusion .	Diagnosis relies on typical immunophenotype (TFE3+/Vimentin+ with absence of myogenic, melanocytic, vascular, and epithelial markers) and molecular confirmation.
Hemangioblastoma	Clear or eosinophilic stromal cells within a richly vascularized stroma.	Vimentin+, S100+, NSE+, α-inhibin+.	TFE3 typically was negative. Site-specific markers (e.g., PAX8) were negative.	Sporadic or associated with VHL syndrome.	Primary pulmonary cases are exceedingly rare; most are metastases. Clear Cytoplasm in CCST-L is likely due to glycogen, not lipid.
Perivascular Epithelioid Cell Tumor (PEComa family), e.g., Pulmonary Clear Cell "Sugar" Tumor	Clear or eosinophilic cells with polymorphic features. PAS stain was positive.	HMB45+, Melan-A+, SMA (focal+), desmin (variable). A subset may express TFE3.	CCST-L lacks expression of myomelanocytic markers (SMA, HMB45, Melan-A, MITF) which are positive in PEComa.	*TSC1/TSC2* mutations or *TFE3* rearrangements.	Significant morphological and immunophenotypic overlap with CCST-L. Absence of myomelanocytic marker expression is key to distinguishing CCST-L.
Epithelioid Hemangioendothelioma (EHE)	Epithelioid endothelial cells with abundant eosinophilic cytoplasm embedded in a myxohyaline or chondromyxoid matrix.	CD31+, ERG+, CD34+, FLI1+. Nuclear CAMTA1+ (in *WWTR1::CAMTA1* rearranged cases). Variable TFE3 expression.	CCST-L is typically CD31-, ERG-.	*WWTR1::CAMTA1* or *YAP1::TFE3* fusions.	Some CCST-L cases show predominantly eosinophilic cytology and diffuse CD34+, mimicking EHE. CCST-L's lack of other vascular markers (CD31, ERG) is critical for distinction.
Sclerosing Pneumocytoma	Biphasic tumor: surface cuboidal cells and stromal round to polygonal cells.	Surface cells: EMA+, TTF-1+. Stromal cells: Vimentin+.	CCST-L is EMA-, TTF-1-.	No specific recurrent fusion.	Overlapping growth pattern, but CCST-L lacks the EMA+/TTF-1+ surface cell population.
Solitary Fibrous Tumor	SFTs are composed of haphazardly arranged spindled to ovoi cells with indistinct, pale eosinophilic cytoplasm within a variabi collagenous stroma, admixed with branching and hyalinize staghorn-shaped blood vessels.	STAT6 (strong nuclear+), CD34+.	TFE3-, and typically negative for S100, etc.	*NAB2::STAT6* gene fusion.	Thoracic SFT is typically pleura-based, while CCST-L occurs within lung parenchyma, often intimately associated with bronchiolar epithelium. STAT6 nuclear positivity is a reliable discriminator.
Metastatic Clear Cell Carcinoma (e.g., Renal Cell Carcinoma, Müllerian Clear Cell Carcinoma)	Clear cells in alveolar, tubular, or solid patterns with rich vasculature.	PAX8+,HNF1β, CA-IX+ (RCC), Napsin A+ (some pulmonary/gynecologic) and other site-specific markers.	TFE3 usually negative (except TFE3-rearranged RCC). CCST-L is PAX8-, CA-IX-.	Varies by primary site (e.g., *VHL* mutations in renal clear cell carcinoma (RCC).	Clinical history and imaging to identify a primary site are essential. Organ-specific IHC markers (e.g., PAX8) are key for confirming metastasis.
Clear Cell Sarcoma	Uniform epithelioid cells with vesicular nuclei, often containing melanin, and rosette-like arrangements around multinucleated giant cells.	S100+, SOX10+, HMB45+, Melan-A+.	CD34-, TFE3-.	*EWSR1::ATF1* or *EWSR1::CREB1* fusions.	Typically arises in deep soft tissues of distal extremities in adolescents/young adults. CCST-L does not express melanocytic markers.
Melanoma (Metastatic)	Highly variable morphology: epithelioid, spindle cells with clear/eosinophilic cytoplasm.	S100+, SOX10+, HMB45+, PNL2, Melan-A+ (variable expression).	TFE3-, and lacks specific gene fusions.	Frequent *BRAF*, *NRAS* mutations.	The most common mimic in metastatic settings. TFE3 negativity and lack of melanocytic markers favor CCST-L, but clinical correlation and molecular testing may be needed for widely metastatic melanoma.

#### 3.6.1 Distinction from primary pulmonary and pleural tumors

Among primary thoracic neoplasms, hemangioblastoma shows histological overlap with CCST-L and demonstrates diffuse IHC expression of Vimentin, S100, NSE, and α-inhibin. However, primary pulmonary hemangioblastoma is exceedingly rare, with most pulmonary cases representing metastases, often associated with von Hippel-Lindau (VHL) syndrome. The clear cytoplasm in CCST-L is more likely attributable to glycogen rather than the lipid accumulation typical of hemangioblastoma (3).

A significant and complex differential diagnosis is the perivascular epithelioid cell tumor (PEComa) family, including pulmonary clear cell “sugar” tumor. There is marked morphological and immunophenotypic overlap, further complicated by the fact that a subset of PEComas harbor *TFE3* rearrangements (including *YAP1::TFE3* in inflammatory spindle cell variants) and can thus show TFE3 protein overexpression (14, 15). The critical discriminator is the consistent absence of myomelanocytic markers (SMA, HMB45, Melan-A, MITF) in CCST-L, which are typically expressed in PEComas.

Epithelioid hemangioendothelioma (EHE) is another important mimic, particularly as some CCST-L cases exhibit predominantly eosinophilic cytology with diffuse CD34 expression. While both tumors may share CD34 positivity and can harbor *YAP1::TFE3* fusions, EHE consistently expresses other definitive vascular markers such as CD31 and ERG, which are absent in CCST-L. The distinctive myxohyaline matrix of EHE is also a useful histological clue.

Sclerosing pneumocytoma can be distinguished by its biphasic population of surface (EMA+/TTF-1+) and stromal cells, a feature absent in CCST-L. Solitary fibrous tumor is composed of haphazardly arranged spindled to ovoid cells with indistinct, pale eosinophilic cytoplasm within a variably collagenous stroma, admixed with branching and hyalinized staghorn-shaped blood vessels. Solitary fibrous tumor is typically pleura-based and exhibits strong nuclear STAT6 and CD34 expression, *NAB2::STAT6* gene rearrangement, contrasting with the STAT6-negative, TFE3-positive profile, *YAP1::TFE3* fusion of parenchyma-based CCST-L.

#### 3.6.2 Exclusion of metastatic disease

In the context of multifocal lung lesions, metastatic tumors become the primary diagnostic concern. The most common mimics include metastatic clear cell renal cell carcinoma (RCC) and Müllerian clear cell carcinoma, which can be identified using organ-specific IHC markers (e.g., PAX8, CA-IX, Napsin A) in conjunction with clinical history and imaging.

Metastatic clear cell sarcoma and melanoma are also critical exclusions. Clear cell sarcoma consistently expresses melanocytic markers (S100, SOX10, HMB45, Melan-A) and harbors *EWSR1::ATF1* or *EWSR1::CREB1* fusions. Melanoma, while morphologically versatile, lacks specific translocations and shows variable melanocytic marker expression. In both, the absence of TFE3 expression and the *YAP1::TFE3* fusion are key distinguishing features from CCST-L.

#### 3.6.3 Role of molecular diagnostics in complex cases

In diagnostically challenging cases with ambiguous morphology or immunophenotype, molecular analysis is definitive. For instance, rare CCST-L cases may be considered in the differential of *GLI1*-fusion-positive mesenchymal neoplasms, with targeted RNA sequencing resolving the diagnosis by identifying *YAP1::TFE3* fusion (3). This underscores that in the modern diagnostic workflow, molecular confirmation remains the ultimate arbiter for CCST-L and its mimics.

### 3.7 Diagnostic approach and technical considerations

#### 3.7.1 Sensitivity and specificity of diagnostic methods

Immunohistochemistry (IHC): Nuclear TFE3 expression was reported in approximately 95% (22/23) of published cases. It serves as a rapid and cost-effective screening tool prior to molecular confirmation. Fluorescence *In Situ* Hybridization (FISH): The *YAP1::TFE3* fusion was confirmed by FISH in about 86% (18/21) of cases. It provides direct genetic evidence and helps rule out IHC false positives. Immunohistochemistry for YAP1 C-terminus (YAP1-CT) and TFE3 serves as a reliable surrogate marker for detecting *YAP1::TFE3* fusions. The combined pattern of YAP1-CT loss with concurrent TFE3 nuclear overexpression demonstrates higher sensitivity than *TFE3* FISH in predicting molecularly confirmed fusions (10). Next-Generation Sequencing (NGS): RNA-based NGS or targeted DNA/RNA panels can definitively identify the *YAP1::TFE3* fusion and concurrently detect other genetic alterations (e.g.,*STED2* mutations), offering a comprehensive molecular profile.

#### 3.7.2 Potential technical pitfalls and solutions

##### 3.7.2.1 IHC false positives/negatives

Immunohistochemistry (IHC) for TFE3, while a highly sensitive screening tool, presents challenges related to both false-positive and false-negative interpretations. Neoplasms with oncogenic TFE3 fusions frequently display aberrant reactivity pattern (strong, diffuse and homogeneous staining in all tumor cells that is comparable to the onslide control) as opposed to the wildtype pattern (usually highly heterogeneous and variable compared to the control) (3). A key diagnostic pitfall is the non-specific nature of nuclear TFE3 positivity, which can also be observed in other neoplasms such as perivascular epithelioid cell tumors (PEComas) and epithelioid hemangioendotheliomas, potentially leading to misdiagnosis. Conversely, false-negative results may occur in CCST-L cases exhibiting weak or focal TFE3 expression. To mitigate these issues, IHC findings must be interpreted within a comprehensive diagnostic framework. This involves correlating staining patterns with classical histomorphology and employing a broader immunohistochemical panel. In this context, diffuse and strong co-expression of vimentin, coupled with negativity for markers such as HMB45, CD31, and STAT6, provides strong supportive evidence for CCST-L. Ultimately, cases demonstrating weak, atypical, or diagnostically ambiguous TFE3 staining should be referred for molecular confirmation to ensure diagnostic accuracy.

##### 3.7.2.2 FISH detection failure and fusion variant limitations

While fluorescence *in situ* hybridization (FISH) is a widely used method for detecting gene rearrangements, its application in diagnosing *YAP1::TFE3* fusion faces two main technical challenges. First, false-negative results may arise from suboptimal sample quality, such as tissues with extensive necrosis or poor fixation, or from probe designs that do not span all potential genomic breakpoints, thereby failing to capture certain fusion variants. To minimize this risk, careful selection of viable, tumor-enriched areas for analysis is essential. In cases with strong TFE3 immunohistochemical expression but negative FISH results, next-generation sequencing (NGS) should be employed as a confirmatory step, given its ability to identify fusion events irrespective of breakpoint location. Second, a more inherent limitation of FISH is its inability to discriminate between different splice isoforms of the *YAP1::TFE3* fusion. This is clinically relevant because certain truncated or alternatively spliced variants may be associated with more aggressive tumor behavior. Therefore, in patients presenting with high-risk features, such as multifocal or metastatic disease, supplemental NGS is recommended not only to confirm the fusion but also to characterize its specific isoform, which may offer important prognostic insights and guide further management.

#### 3.7.3 Integrated diagnostic strategy and clinical recommendations

**Figure 2:**
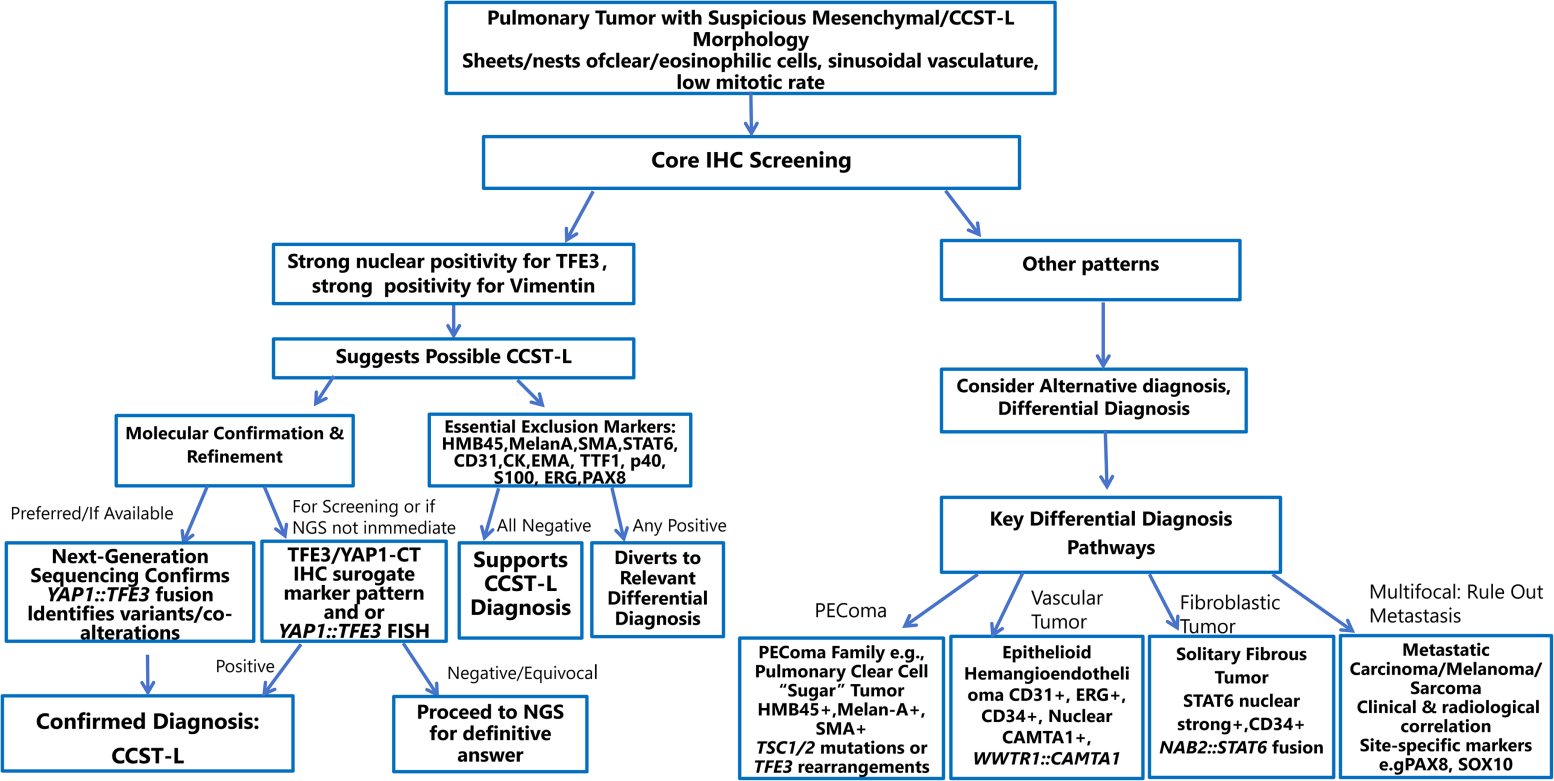
Comprehensive diagnostic and differential diagnostic decision algorithm for Clear Cell Stromal Tumor of the Lung (CCST-L). This flowchart provides pathologists with a clear, step-by-step guide from initial morphological suspicion to final molecular confirmation of CCST-L. It integrates key histological features, immunophenotypic analysis, molecular testing strategies, and critical distinctions from major mimics, emphasizing a multimodal, evidence-based diagnostic approach.

The diagnosis of CCST-L follows a stepwise, multimodal approach that begins with histomorphologic suspicion and culminates in molecular confirmation. A comprehensive diagnostic algorithm integrating these steps is provided in **Figure 2**.

The diagnostic pathway is initiated for pulmonary neoplasms exhibiting characteristic morphology, including sheets or nests of clear to eosinophilic cells within a sinusoidal vascular network and low mitotic activity. Immunohistochemistry (IHC) plays a dual pivotal role. First, as a screening tool, nuclear TFE3 positivity (noting its high sensitivity but potential for false positives) coupled with diffuse, strong vimentin co-expression provides a crucial initial clue toward CCST-L. Second, and equally critical, is the systematic use of an exclusion IHC panel, including CK, HMB45, Melan-A, SMA, STAT6, CD31, ERG, TTF-1, Napsin A, HNF 1β and PAX8, to definitively rule out key histological mimics such as PEComa, vascular tumors (e.g., epithelioid hemangioendothelioma), solitary fibrous tumor, and metastatic carcinomas.

Molecular confirmation remains the diagnostic cornerstone. The gold standard is RNA-based next-generation sequencing (NGS), which definitively identifies the *YAP1::TFE3* gene fusion. Surrogate and complementary tests include the highly specific IHC pattern of YAP1 C-terminal loss with concurrent TFE3 nuclear overexpression, and YAP1::TFE3 fluorescence *in situ* hybridization (FISH), the latter of which carries a risk of false negatives and cannot discriminate between fusion isoforms. NGS is strongly indicated for all suspected cases, particularly those with an atypical immunophenotype, diagnostic difficulty, or negative FISH results, as it provides definitive diagnosis and can identify prognostically relevant fusion variants.

Special clinical and logistical scenarios require tailored approaches. In settings with limited access to molecular tests, a confident diagnosis can be established using a strict morphology-driven IHC approach, provided all criteria(characteristic histology, TFE3/vimentin co-expression, and negativity for the full exclusion marker panel) are met, while explicitly acknowledging the limitations of this method. For patients presenting with multifocal disease, metastatic tumors must be rigorously excluded first through close integration of clinical history, imaging studies, and an expanded IHC panel. Finally, for suspected cases that test negative for the *YAP1::TFE3* fusion, comprehensive NGS profiling is recommended to search for alternative genetic drivers, thereby preventing the misdiagnosis of rare CCST-L subtypes or other novel entities.

### 3.8 Treatment and clinical outcomes

#### 3.8.1 Current therapeutic approaches and outcomes

No standardized treatment guidelines exist for CCST-L due to its extreme rarity. Analysis of aggregated case data reveals the following:

##### 3.8.1.1 Primary therapy

Complete surgical resection (lobectomy or wedge resection) constitutes the mainstay of management for localized disease and is regarded as potentially curative.

##### 3.8.1.2 Outcomes in resected cases

Among patients who underwent resection with available follow-up data (n=24), the majority (19/24, 79.2%) remained disease-free over a median follow-up period of 38 months, supporting the premise of an often indolent clinical course following complete excision.

Management of Advanced Disease: For patients with unresectable multifocal or metastatic disease, management has been heterogeneous and largely suboptimal, encompassing chemotherapy or best supportive care. Outcomes in this subgroup are poor, underscoring the critical lack of effective systemic therapeutic options.

#### 3.8.2 The clinicopathological spectrum: identifying aggressive disease

It is imperative to recognize that CCST-L exhibits a biological spectrum, and a distinct subset pursues an aggressive clinical course. Approximately 23.3% (7/30) of reported patients developed documented metastasis or progressive disease (3, 8, 11). Multifocality at presentation, particularly bilateral lung involvement, emerges as a strong clinical indicator of this aggressive phenotype (8, 11). Histologically, most aggressive cases do not display overtly high-grade morphology. Commonly observed features include a prominent stromal inflammatory infiltrate (lymphocytes, plasma cells, histiocytes) and, in some instances, geographic necrosis. The exceptional fatal case reported by Dehner et al. demonstrated geographic necrosis and unequivocally elevated mitotic activity (6 per 2 mm²) (11).

#### 3.8.3 Clinical recommendations and unmet needs

Necessity for Long-Term Surveillance: Given the documented potential for late recurrence and metastasis, prolonged, likely lifelong, clinical and radiographic follow-up is strongly recommended for all patients diagnosed with CCST-L, irrespective of the initial disease stage.

Toward a Standardized Management Strategy: Existing evidence highlights the urgent need to establish consensus-driven management guidelines. These should address the role of adjuvant therapy in high-risk scenarios and create a framework for evaluating systemic agents in advanced disease.

Future Research Imperative: International multi-institutional collaboration is paramount to accrue sufficient data on advanced cases. This effort is essential to identify reliable predictive biomarkers and to facilitate the development of meaningful clinical trials for metastatic CCST-L.

## 4 Discussion

### 4.1 Underlying molecular mechanisms

Aggressive cases have been molecularly confirmed to harbor the canonical *YAP1::TFE3* fusion, indicating that tumor aggressiveness is not exclusively defined by a unique fusion variant but likely involves additional genetic or microenvironmental modifiers, such as a *STED2* mutation of uncertain significance (3). Key contributing mechanisms may include as follows.

#### 4.1.1 Primary oncogenic driver enhancement

The constitutive activation driven by the *YAP1::TFE3* fusion oncoprotein is central to tumorigenesis in CCST-L. YAP1, a core effector of the Hippo pathway, regulates cell proliferation and anti-apoptosis, while TFE3 is a transcription factor that activates genes involved in lysosomal biogenesis and angiogenesis (16). The chimeric protein resulting from their fusion amplifies both oncogenic functions, directly driving hyperproliferation, conferring early migratory capacity, and conferring inherent resistance to apoptosis. This foundational mechanism can be further potentiated by secondary events and the tumor microenvironment, which likely underpin the aggressive behavior observed in cases with multifocal or metastatic disease.

#### 4.1.2 Secondary genomic events

In addition to the primary fusion event, secondary genomic alterations may modulate tumor behavior. The current evidence for such events in CCST-L is emerging, and some mechanisms are proposed based on analogies with other tumor types.

##### 4.1.2.1 Fusion gene splice variants

Specific splice isoforms of the *YAP1::TFE3* fusion, for instance, short variants linking *YAP1* exons 1–4 to *TFE3* exons 6–10 may exhibit stronger transcriptional activity, leading to more potent activation of pro-metastatic genes such as *VEGF* and *matrix metalloproteinases* (MMPs) (17), thereby promoting multifocal growth and distant metastasis.

##### 4.1.2.2 Copy number variations

Inferred from the genomic landscape of aggressive sarcomas, it is plausible that highly aggressive CCST-L cases may harbor amplifications or deletions in key genomic regions such as *MYC*, *PTEN*, or *TP53*, which could enhance proliferative drive or genomic instability. However, systematic profiling to confirm this in CCST-L is awaited.

##### 4.1.2.3 Co-occurring mutations

By analogy with other cancers driven by transcription factor fusions, secondary mutations in genes like *PIK3CA* or *BRAF* could theoretically further activate pathways such as PI3K-AKT-mTOR, potentially augmenting anti-apoptotic signaling and migratory capacity. Their presence and role in CCST-L progression remain to be investigated.

#### 4.1.3 Role of the tumor microenvironment

##### 4.1.3.1 Stromal activation

The *YAP1::TFE3* fusion protein can induce tumor cells to secrete high levels of TGF-β and PDGF, leading to hyperactivation of cancer-associated fibroblasts (CAFs) (18). Activated CAFs remodel the extracellular matrix (ECM) by excessive collagen deposition, creating physical “tracks” for tumor invasion. In synergy with the fusion oncoprotein, CAFs also upregulate VEGF, promoting angiogenesis and lymphangiogenesis, thereby facilitating hematogenous and lymphatic spread (e.g., to hilar lymph nodes, liver, or kidneys).

##### 4.1.3.2 Immune-suppressive niche

YAP1 activation directly upregulates PD-L1 expression on tumor cells, contributing to immune evasion by inhibiting cytotoxic T-cell activity. This allows tumor cells to escape immune surveillance and establish distant metastases (16).

##### 4.1.3.3 Cytokine and chemokine dysregulation

Elevated TGF-β not only enhances tumor cell motility but may also guide directional migration to specific distant organs (e.g., liver and kidney), as illustrated by the reported case of a 29-year-old female with renal and hepatic involvement.

##### 4.1.3.4 ECM remodeling and mechanotransduction

Through the synthesis of ECM proteins and induction of stromal remodeling, the fusion protein participates in aberrant mechanotransduction, fostering a tumor-promoting feedback loop between the malignant cells and their microenvironment (18).

These proposed mechanisms highlight potential therapeutic targets, including Hippo pathway inhibitors, immune checkpoint blockade (e.g., anti-PD-1/PD-L1), and agents targeting stromal activation or specific secondary mutations. These proposed mechanisms highlight potential therapeutic targets, including Hippo pathway inhibitors, immune checkpoint blockade (e.g., anti-PD-1/PD-L1), and agents targeting stromal activation or specific secondary mutations.

#### 4.1.4 Implications of fusion-negative cases: a spectrum of possibilities

The existence of fusion-negative cases highlights key unknowns in CCST-L biology and presents several non-mutually exclusive possibilities.

##### 4.1.4.1 Alternative genetic drivers

They may represent tumors driven by alternative, yet-to-be-identified genetic alterations. This could involve fusions with other *TFE3* partners or mutations in distinct pathways that converge to produce a similar histomorphological and immunophenotypic endpoint.

##### 4.1.4.2 Non-canonical TFE3 activation

They could indicate alternative mechanisms leading to TFE3 protein overexpression without a canonical gene fusion, such as dysregulation of upstream signaling pathways, epigenetic modifications, or abnormalities in protein degradation.

##### 4.1.4.3 Molecular heterogeneity

Most importantly, these cases underscore the potential molecular heterogeneity within tumors currently classified as CCST-L based on core morphology and immunoprofile. This heterogeneity highlights a critical area for future research, where comprehensive molecular profiling (e.g., whole-transcriptome sequencing) of such cases is essential to define potential novel subtypes and complete the molecular taxonomy of this rare entity.

### 4.2 Data limitations

#### 4.2.1 Data limitations

Comprehensive analysis of all 30 reported cases-integrating clinical presentation, imaging, nodal/metastatic status, histopathology, and outcomes-demonstrates that CCST-L exhibits a clinicopathological spectrum ranging from indolent to locally aggressive/metastatic disease. It is important to note the following limitations of this study.

##### 4.2.1.1 Insufficient sample size and representativeness

Only 30 cases were identified from 11 published studies, which constitutes an extremely limited sample. This small cohort may not adequately capture the full heterogeneity of the disease, potentially leading to inaccurate estimations of population incidence, true aggressive potential, and distinct subtype characteristics.

##### 4.2.1.2 Incomplete follow-up data

Follow-up information was unavailable for six cases. Follow-up information of 24 patients was available and for nine patients, follow-up duration was limited to 3–12 months. This timeframe is likely shorter than the potential recurrence interval, particularly for low-grade tumors, thereby introducing bias into the assessment of long-term outcomes and recurrence patterns.

##### 4.2.1.3 Missing clinical data and heterogeneity

Key clinical details-including presenting symptoms, specific imaging features were inconsistently reported across studies. Substantial variability in management approaches (e.g., surgery vs. biopsy plus chemotherapy) further precluded standardized analysis of treatment efficacy.

##### 4.2.1.4 The role of *YAP1::TFE3* as a diagnostic marker

The diagnostic utility of this fusion is nuanced by a subset of morphologically typical but fusion-negative tumors (3, 5, 11). The *YAP1::TFE3* fusion-negative tumors may be driven by alternative genetic alterations, such as fusions with other *TFE3* partners or mutations in distinct pathways, representing yet undefined molecular subtypes that phenocopy the canonical entity. TFE3 protein overexpression may occur via non-canonical, non-genetic mechanisms, such as dysregulation of upstream signaling or epigenetic modifications, indicating that activation of the TFE3 pathway can occur independently of the defining fusion. Consequently, while the *YAP1::TFE3* fusion is a highly characteristic molecular feature, its interpretation must be integrated with histomorphological and immunohistochemical findings. Its universal diagnostic validity awaits further validation in larger cohorts.

##### 4.2.1.5 Limited mechanistic insights

While the central role of the *YAP1::TFE3* fusion gene is established, systematic investigations into secondary genomic events, tumor microenvironment interactions, and other regulatory mechanisms are lacking. This gap hinders a clear biological explanation for the observed variability in tumor aggressiveness. In addition, the existence of fusion-negative tumors highlights key unknowns and present several non-mutually exclusive possibilities: (i) Alternative Genetic Drivers (e.g., other *TFE3* fusions or distinct pathway mutations); (ii) Non-Canonical TFE3 Activation (e.g., via epigenetic dysregulation); and (iii) underlying Molecular Heterogeneity. This gap underscores the need for systematic studies to delineate alternative oncogenic pathways.

#### 4.2.2 Need for multi-institutional collaboration

To overcome these limitations, we emphasize the critical need for large-scale, multi-institutional collaboration. Such efforts are essential to fully characterize the clinicopathological and molecular spectrum of CCST-L. Such collaborative efforts can: (i) aggregate cases across centers to build a larger, more representative cohort for analysis; (ii) establish standardized protocols for collecting clinical, pathological, and follow-up data, thereby reducing heterogeneity and information bias; and (iii) consolidate technical and intellectual resources to systematically investigate underlying molecular mechanisms and evaluate potential therapeutic approaches, ultimately accelerating translational progress in this rare disease.

### 4.3 Synthesis: linking molecular heterogeneity to clinical behavior

The molecular underpinnings of CCST-L likely form a continuum that correlates with its clinical spectrum. The canonical *YAP1::TFE3* fusion provides a foundational oncogenic signal. Indolent behavior may be sustained by this primary driver alone or with minimal modifying events. In contrast, aggressive and metastatic phenotypes may arise through a confluence of mechanisms: the acquisition of specific, more potent fusion isoforms (e.g., certain splice variants); the superposition of secondary genetic hits (e.g., CNVs affecting *MYC* or *TP53*, or co-occurring mutations); and/or a permissive or actively promoting tumor microenvironment characterized by immune evasion and stromal remodeling. The reported *STED2* mutation in an aggressive, fusion-negative case further underscores that alternative pathways can converge on a similar aggressive phenotype. Thus, the clinical heterogeneity of CCST-L probably mirrors its underlying molecular heterogeneity, where the net oncogenic output is determined by the interplay of primary drivers, secondary genomic events, and microenvironmental cues.

### 4.4 Future directions

Moving forward, we propose several actionable directions.

Clinical Research. (i) Multicenter retrospective cohort studies. To evaluate the impact of surgical extent (lobectomy vs. wedge resection) on patient outcomes, and to define intervention thresholds for high-risk features such as multifocality and metastasis. (ii) Targeted therapy trials. To design and conduct clinical trials evaluating agents that inhibit the *YAP1::TFE3* pathway, such as Hippo signaling inhibitors. (iii) Therapeutic optimization in advanced disease. To explore the optimization of chemotherapy regimens, including combination strategies with targeted agents, and to clarify the role of systemic therapy in late-stage CCST-L.

Basic Research. (i) Functional characterization of *YAP1::TFE3* fusion variants. To investigate the biological differences among alternative splicing isoforms of the *YAP1::TFE3* fusion, and their potential synergistic effects with secondary genomic alterations such as *MYC* and *Notch* amplification. (ii) Tumor microenvironment and immune evasion. To explore the role of PD-L1-mediated immune escape mechanisms within the tumor microenvironment. (iii) Identification of alternative oncogenic drivers. To systematically screen for additional driver events, such as *STED2* mutations, and characterize their associated signaling pathways.

## 5 Summary

In summary, our review of the literature confirms that CCST-L is a distinct tumor entity characterized by diffuse immunohistochemical expression of TFE3 and Vimentin, and with a *YAP1::TFE3* gene fusion. The presence of multiple bilateral pulmonary lesions, lymph node involvement, and extrapulmonary metastases are identified as risk factors for CCST-L. International multicenter collaboration is essential to accumulate a larger case series, which will facilitate a more comprehensive understanding of its clinicopathological features, optimize treatment strategies, refine prognostic assessment, and ultimately improve clinical management.
