# Correction: Acomprehensive review of clear cell stromal tumor of the lung: integrating clinicopathology and molecular pathogenesis

**DOI:** 10.3389/fonc.2026.1931508

**Published:** 2026-08-05

**Authors:** Liping Luo, Liang Lv, Ying Zeng

**Affiliations:** 1 Department of Pathology, Daping Hospital, Army Medical University, Chongqing, China; 2 Department of Pathology, Chongqing Public Health Medical Center, Chongqing, China; 3 Department of Pathology, The Thirteenth People’s Hospital of Chongqing, Chongqing, China

**Keywords:** clear cell stromal tumor of the lung, clinicopathology, molecular pathogenesis, treatment and prognosis, *YAP1::TFE3* gene fusion

The **Author contributions** statement has been corrected to read:

“LPL: Writing – original draft. LL: Writing – original draft, Conceptualization, Data curation. YZ: Writing – review & editing.”

Author Liang Lv was erroneously omitted as a co-first author and equal contributor alongside Liping Luo.

The original version of this article has been updated.

